# Four-Dimensional (4D) Printing of Dynamic Foods—Definitions, Considerations, and Current Scientific Status

**DOI:** 10.3390/foods12183410

**Published:** 2023-09-13

**Authors:** Ahmed Raouf Fahmy, Antonio Derossi, Mario Jekle

**Affiliations:** 1 Department of Plant-Based Foods, Institute of Food Science and Biotechnology, University of Hohenheim, 70599 Stuttgart, Germany; ahmedraouf.fahmy@uni-hohenheim.de; 2 Department of Agriculture, Food Natural Resources and Engineering (DAFNE), University of Foggia, 71122 Foggia, Italy; antonio.derossi@unifg.it

**Keywords:** 4D food printing, 3D printing, smart materials, shape morphing, programmed structures

## Abstract

Since its conception, the application of 3D printing in the structuring of food materials has been focused on the processing of novel material formulations and customized textures for innovative food applications, such as personalized nutrition and full sensory design. The continuous evolution of the used methods, approaches, and materials has created a solid foundation for technology to process dynamic food structures. Four-dimensional food printing is an extension of 3D printing where food structures are designed and printed to perform time-dependent changes activated by internal or external stimuli. In 4D food printing, structures are engineered through material tailoring and custom designs to achieve a transformation from one configuration to another. Different engineered 4D behaviors include stimulated color change, shape morphing, and biological growth. As 4D food printing is considered an emerging application, imperatively, this article proposes new considerations and definitions in 4D food printing. Moreover, this article presents an overview of 4D food printing within the current scientific progress, status, and approaches.

## 1. Introduction

The general interest in additive manufacturing (AM) is mainly based on its principle of operation where large structures are built in layer-based and rapid prototyping (RP) techniques. The combination of RP and layer-based fabrication provides superior accuracy and precision compared to traditional processing and structuring techniques [1,2,3,4]. For the structuring of foods, the adoption of 3D printing technologies was initially motivated by design possibilities that are either challenging or cannot be achieved using traditional food processing techniques. However, through comprehensive scientific investigations, the application of 3D printing technologies shifted towards an increase in consumer acceptance by the functional control over food structures in terms of nutrition, flavor, and textural properties.

Three-dimensional printing is a rapid prototyping technique for processing foods where 3D structures are built using a layer-based computer-aided design (CAD) approach [5]. Processing food structures using 3D printing involves multiple sequential stages. First, food constructs are modeled and designed using CAD software. Second, the model is uploaded into slicing/printing software where the material–process-dependent settings are applied. The slicing software is responsible for applying the printing settings and slicing the geometrical model for the layer-based deposition of the material. Third, through the slicing software, a G-code is generated and processed by the printer where the designed geometries are structured. Finally, a post-processing step in the form of thermal stabilization is usually applied (material-dependent) for the consumption or storage of 3D-structured foods.

**Figure 1:**
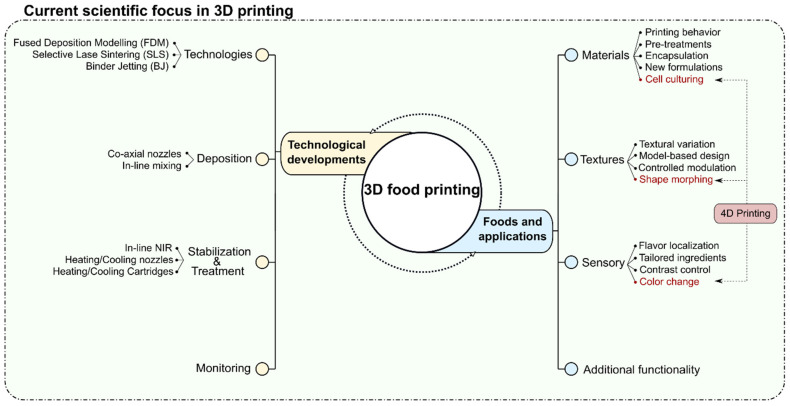
Schematic representation of the current research in 3D food printing.

Currently, the research and investigations in the field of 3D food printing can be divided into two main categories, as shown in Figure 1, which are technological developments and applications in food. During its early adoption and development period, 3D food printing research was mainly focused on the printing applicability of different food formulations by elucidating the material–process interactions [6,7,8,9,10]. From a technological perspective, the intensive investigations into the relationship between material behavior and 3D printing led to the development of technological solutions in process monitoring, design and structuring, complementary methods (such as in-line mixing, liquid injection, and co-axial extrusion), and post-processing/stabilization techniques. Also, the native form of different food materials (powders, liquids, and solids) has motivated the adoption of different 3D printing technologies such as fused deposition modeling (FDM), binder jetting (BJ), and selective laser sintering (SLS) [11,12,13]. Consequently, technological developments have broadened the application range of 3D food printing for the structuring of customized food textures, precise localization of flavors, printing of anisotropic protein structures, and processing of food side streams, with a focus on the application of personalized nutrition in the near future [14,15,16,17,18,19,20,21,22,23,24].

Recently, 3D printing has been utilized to process food structures that exhibit time-dependent behaviors, which is defined as 4D food printing. The main aim of using a 4D printing approach in food science is to generate 3D food structures with programmable changes in appearance and functionality with time and upon stimulation. To illustrate, programmable changes in functionality are described as pre-defined (using material tailoring or specific geometrical designs) food transformations affecting nutritional and sensory aspects. As an example, chemical and thermal stimulations are used to achieve a color change or the shape morphing of printed food structures [25,26]. Since 4D food printing is an emerging processing approach, this article defines 4D food printing from a scientific perspective and introduces specific considerations for this approach. In the following, we present the scientific progress and state-of-the-art applications in 4D food printing according to the established definitions and considerations.

## 2. 4D Printing: Considerations and Definitions

Additive manufacturing or the 3D printing of engineering and food structures is the repetitive planar structuring of materials in a layer-based approach. To illustrate, the planar structures are overlaid to create a static 3D structure with geometrical features that are determined by a pre-defined design. In addition, its bulk materials and mechanical properties are defined by the applied process, processing parameters, and the material ingredients and interactions. By mentioning that processing using a 3D printing approach leads to the creation of static structures, it is implied that the functionalities and overall properties of the additively processed engineering or food materials and structures are time-independent. As a direct integral application of additive manufacturing, 4D printing should be considered as an extension of 3D printing [27]. Three-dimensional (3D) printing is the processing of structures in the three spatial coordinates: the x-, y-, and z-axes. To emphasize, 4D printing introduces a further dimension to 3D-printed structures, which is time [28,29,30,31]. Therefore, 4D printing uses a 3D printing approach to process dynamic structures with properties that can vary with time. Generally, the dynamism and time-dependent behavior of 4D-processed structures are a result of smart structural design and tailored material compositions in combination with the application of several stimuli [32,33,34]. The induction of this controlled or programmed time-dependent behavior is associated with the use of chemical stimulus [35,36,37,38,39] or thermal stimulus [40,41]. Moreover, the time-dependent behavior of 4D-printed structures is referred to as programmed or controlled changes, indicating that targeted transformations are pre-defined, intentional, and highly desired. Similar to 3D printing, the development of 4D printing was performed for engineering applications before its adoption for food structuring. The approach was mainly motivated by the creation of smart materials and structures that can alter their functionality and micro/macrostructural behavior under different stimuli with respect to time.

Since the conception of 4D food printing, different definitions have emerged along with different extensions (5D or 6D). This is indicative of the absence of a consensus on the definition of 4D printing, which is also the cause for the free use of extra dimensions. The visible universe exists in the space-time continuum, and thus, the maximum dimensions that must be considered in printing are four dimensions (three spatial dimensions and a single time dimension). This constitutes that all time-dependent material or structural transformations of printed structures are bound by the time dimension (fourth dimension). From a processing point of view, 5D and 6D printing is the extension of the construction method to include 1 or 2 rotational axes within the printers’ functionality [42]. However, by presenting rotational possibilities in printing, this becomes a definition of degrees of freedom (DOF) and not extra dimensions. Furthermore, in the view of this article, the combination of several time- or stimulation-dependent effects is still and should always be considered as 4D food printing. As an example, a study by Chen et al. presents an approach for the stimulated color change of food structures through probiotic growth [26]. The authors incorporated two time-dependent transformations in 3D-printed food structures and considered this approach as 5D printing. However, this application is still 4D printing from the perspective of our definition, as both effects are bound by the time domain. In addition, for the traditional processing and 3D printing of food materials, thermal stabilization is considered an intrinsic process to achieve specific thermal transitions, including texture (without shape morphing), flavor development, and nutritional changes. Some studies consider effects such as 4D food printing [43]; however, by using this analogy, all thermally treated foods should be considered 4D foods. Thus, texture, flavor development, and nutritional changes through thermal stabilization should not be considered an application of 4D food printing.

Based on the previous discussion, we define 4D food printing as the structuring of foods with one or more programmable and controlled time-dependent transformations (e.g., sensory and nutritional properties) using 3D printing approaches. Transformations of 4D-printed foods are made possible using either external or internal stimuli and cannot be typical transformations of traditionally processed or 3D-printed foods. Therefore, according to the definition, the following rules are applied:

Four-dimensional food structures are processed completely using 3D printing approaches.

The printing of structures with several time-dependent transformations is only 4D food printing without the addition of extra dimensions.

## 3. Programmed Functionality in Printed Food Structures

**Figure 2:**
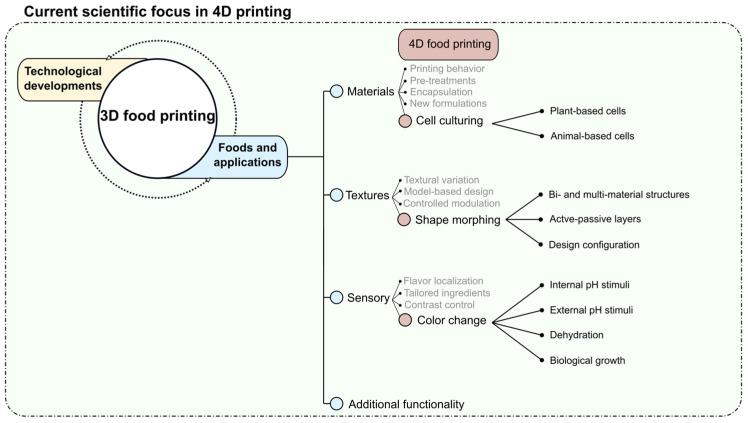
Schematic representation of the current research on 4D food printing.

To highlight the state-of-the-art research and development of 4D food printing, the Scopus, Science Direct, and Google Scholar databases were used for the literature search in this review. Regarding the inclusion criteria, published articles focusing on 4D food printing, triggered food transformations, shape morphing, and color and texture changes were considered. The search strategy led to the inclusion of 34 published articles in the 4D food printing domain. Currently, as shown in Figure 2, there are three main research directions or topics in 4D food printing that are reported in the published articles, including (1) stimulated color change; (2) stimulated shape morphing; (3) cell culturing. For the mentioned research directions, the predominant objective is to achieve programmable food transformations directed towards optimization, esthetics, and most importantly, food functionality. The term programmable indicates the predetermination of specific transformations maintaining a high level of control over time-dependent transitions, which is discussed in the following sections of this review. The concept of using a 4D approach in food applications, to the best of our knowledge, was first introduced by Wang et al. during the proceedings of the Conference on Human Factors in Computing Systems [44]. The authors introduced the shape morphing of printed food structures through hydration-responsive food substrates. From a consumer perspective, this early approach was proposed for processing compact 2D food segments that can be transformed into 3D structures upon cooking, which saves space during packaging, storage, and shipping [44]. From the application and market perspectives, the 4D printing approach broadens the use of technology for creating appealing food products with a wide range of customization, such as the introduction of variable color gradients in foods and heat-dependent variable food shapes and textures [25,45].

### 3.1. Stimulated Color Change

Time- and stimulation-driven color change is one of the most studied topics in the 4D food printing research. Such a high trend in scientific investigations is driven by two main reasons. First, the implementation of color-changing kinetics in 3D-printed structures is relatively simple compared to other 4D printing applications, such as shape morphing and cell culturing. To illustrate, the printing of color-shifting structures is driven by the introduction of plant-based bioactive compounds, which are pH stimuli-responsive inside printable food materials. The commonly introduced plant-based bioactive compounds are anthocyanins, carotenoids, betalains, and curcumin [46,47,48,49]. As an example, in alkaline conditions, anthocyanin and curcumin exhibit green and red colors, respectively. On the other hand, in acidic conditions, they exhibit red and yellow colors, respectively. The second reason for the immense focus on 4D-stimulated color change is that the color of food systems is an imperative deterministic factor in the sensory perception and consumer acceptance [50]. Currently, the research and application of stimulated color change in the 4D printing literature can be categorized into four main categories of stimulation [26,49,51,52,53,54,55,56,57,58]:

(1) Mass diffusion—internal pH stimuli;
(2) Post-processing—external pH stimuli;
(3) Post-processing—dehydration;
(4) Microbiological metabolism—internal pH stimuli.

In the application of mass diffusion, internal pH stimuli are mostly performed by the printing of multi-material composite structures. Typically, two or more materials with different hydration levels, pH conditions, and pH-responsive compounds are printed within the same structure. For reaching a pH equilibrium within the printed structure, time- and material-dependent diffusion occurs within the system, thus inducing color change. Regarding the application of post-processing, spraying with different pH solutions or dehydration methods are used to induce a spontaneous color change of the object. Dehydration methods are usually used for moisture transport, which can enhance the alkalinity of the material [55]. The fourth category using microbiological metabolism as a stimulation method, which we categorized earlier, is the most promising application for color change in terms of functionality. In this application, time-dependent biological growth or metabolism of microorganisms (summarized as fermentation) is used to alter the pH, thus changing the printed structure’s color [26]. At the same time, this technique can be used as an indicator of the state of cellular growth inside printed structures.

**Figure 3:**
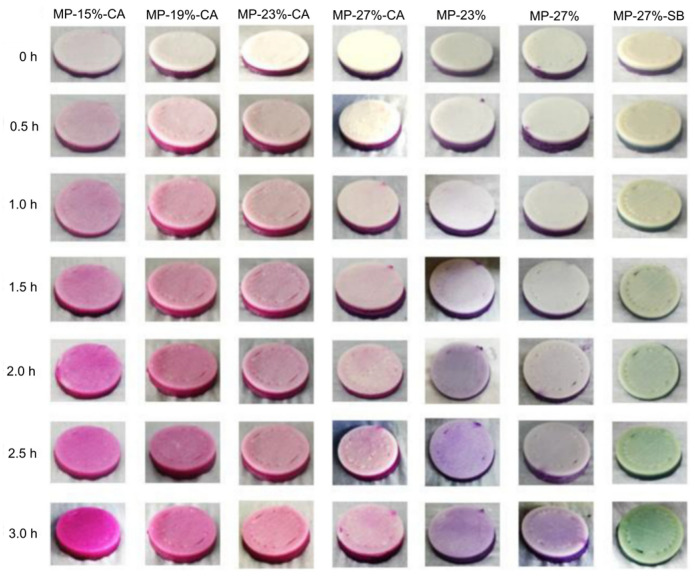
Time-dependent color change of printed dual-material structures composed of mashed potato and anthocyanin-containing purple sweet potato at different pH levels (reproduced from [54] with permission from Elsevier, 2020).

**Figure 4:**
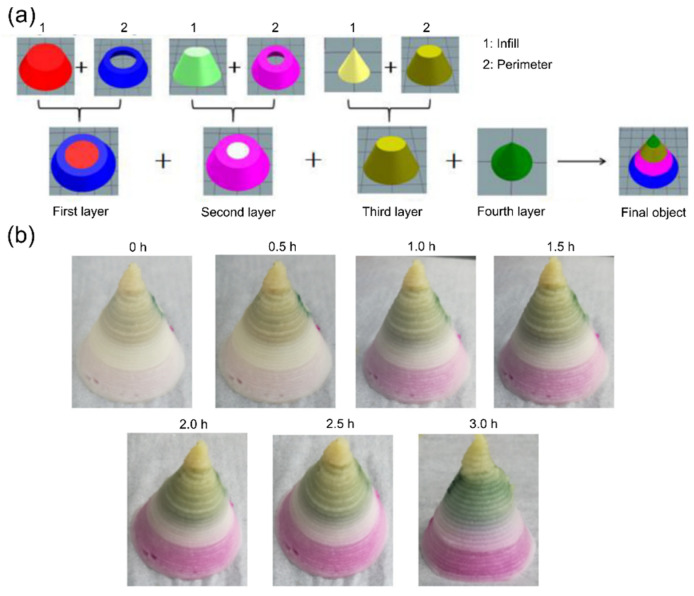
Four-dimensional printing of composite structures with purple sweet potato infill and mashed potato perimeter at different pH levels (reproduced and adapted from [54] with permission from Elsevier, 2020): (**a**) dual extrusion 3D printing model; (**b**) time-dependent mass diffusion and color change.

A pH-stimulated color change in the form of mass diffusion is the most studied 4D printing effect in the scientific literature. For this application, some studies have utilized extracted pH-sensitive pigments, such as anthocyanins and curcumin from plant-based sources in the printed food material systems [52,55,56]. However, most scientific investigations are performed by the 3D printing of ingredients that are natively rich with pH-responsive bioactive compounds, such as beetroot, purple sweet potato, and red cabbage [49,54]. Then, the time-dependent color change is stimulated by either spraying the printed structures with solutions prepared at different pH levels or inducing pH change by printing composite structures, which contain other materials with different pH levels. As an example, He, Zhang, and Guo (2020) investigated the color change of 3D composite structures by printing dual- and multi-materials [54]. For printing dual-material structures, a first phase of anthocyanin-containing purple sweet potato (PSP) and a second phase, with variable pH levels, of mashed potato (MP) were stacked into a disk-shaped configuration. For preparing the MP material phase at different pH levels, the authors used 1% of citric acid (CA) and 1% sodium bicarbonate (SB) of the total weight to achieve acidic (pH ≈ 2.5) and weakly alkaline (pH ≈ 7.8) phases, respectively. Without the addition of CA or SB, the MP phase was in a nearly neutral condition (pH ≈ 6.5). In addition, the authors varied the hydration level of the MP phase by changing the concentration of the potato flakes (15%, 19%, 23%, and 27% based on the weight of water) in the material system. For the printing of the disk-shaped structures, as shown in Figure 3, the PSP phase was located on the bottom, while the variable MP phase was located on the top. The results show that the structures exhibited a time-dependent color change with an increase in the color intensity depending on the MP hydration level and the pH conditions. The color change behavior occurred due to the diffusion of the anthocyanins from the PSP phase to the MP phase. As shown in Figure 3, with the increase in the MP concentration, the color intensity decreased at different times due to the slower diffusion rates. For further consideration, such an observed behavior was also dependent on the area of the diffusion boundary and the hydration discrepancy between the used material systems. Based on the results of the dual-material systems, the authors chose three MP phases (with variable hydration and pH levels) to obtain a higher color change rate for printing multi-material composites. As shown in Figure 4a, four-section composite structures were printed, consisting of PSP infill and variable pH/hydration MP perimeters. Through this approach, the study showed that depending on the distribution of the MP phase and the surface area of the diffusion boundary, the time-dependent color change kinetics can be modulated, as shown in Figure 4b. The study presented a promising example of the 4D processing of complex food structures according to the defined definition in Section 2. The color-changing effects shown in Figure 4 display a pre-defined and controlled transformation that is extrinsic to traditionally processed foods or typical 3D-printed food structures.

Stimulated color changes of 3D-printed structures, as discussed in the previous section, can be used as a primary and programmed effect to alter the perception of foods. However, this 4D printing application can also be used as a secondary effect, such as an indication of other progressing behavior, like time-dependent organic growth or fermentation processes. Several studies have focused on investigating and utilizing pH-driven color changes to monitor the microbiological metabolism in printed structures [26,59]. Chen et al. investigated the color change behavior through the incorporation of probiotics in 3D-printed food structures [26]. In their study, the authors incorporated *Lactobacillus bulgaricus* and *Streptococcus thermophilus* (lyophilized powder form) in a purple sweet potato-based material system. In addition, four different mass concentrations (0.1, 0.2, 0.3, 0.4, and 0.5%) of tea polyphenols were added to the material system to promote metabolism. Moreover, the study investigated time-dependent color changes during fermentation at 37 °C with and without the addition of the polyphenols. During incubation, the number of probiotics reached its maximum value and was saturated between 6 and 8h. Concerning the addition of tea polyphenols, the results showed that increasing the concentration of polyphenols up to 0.4% promoted the metabolism of probiotics. Most importantly, the printing and incubation results showed a significant color change for all printed formulations, from a dark purple value (after printing) to a bright red (after 10 h of fermentation). The time-dependent color shift occurred due to a reduction in the pH as a consequence of the production of lactic acid during fermentation. The authors presented this effect as 5D printing by combining color change and biological growth in one printed system. However, according to our definition, the combination of two time-dependent (single time domain) effects still constitutes a 4D food printing application.

**Figure 5:**
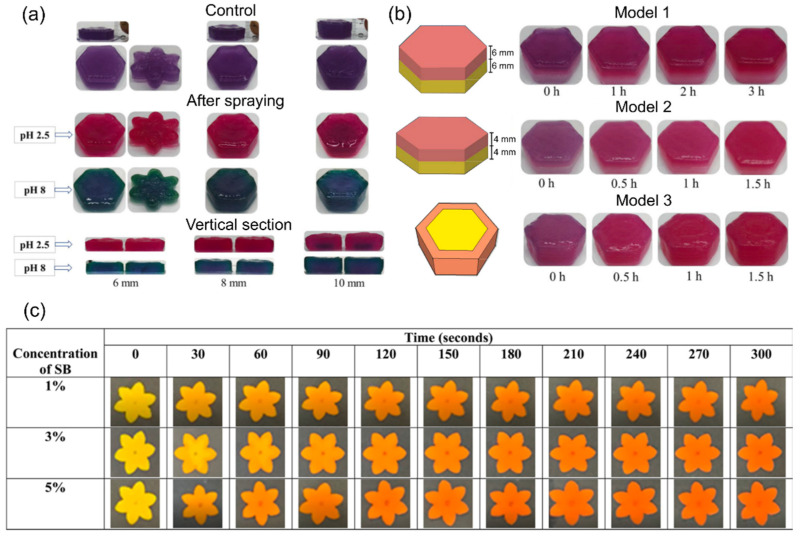
Effects of external and internal pH stimuli on the time-dependent color change behavior of printed structures: (**a**) color change of structures after spraying with solutions at different pH levels (2.5 and 8) after 1.5 h in relation to the structures’ thickness (reproduced from [53] with permission from Elsevier, 2021); (**b**) time-dependent color change of composite structures due to diffusion (reproduced and adapted from [53] with permission from Elsevier, 2021); (**c**) time-dependent color change of single-material structures after immersion in solutions with different concentrations of sodium bicarbonate (reproduced from [51] with permission from American Chemical Society, 2021).

Investigating the effect of post-processing in the form of external pH stimuli, Shanthamma et al. studied the color change behavior of curcumin-based 3D-printed structures [51]. The investigation was performed on a sago flour-based material system with incorporated turmeric powder at different concentrations of 0, 0.5, 1.5, and 2.5% on a weight basis. To stimulate the time-dependent color change effect, the authors used a post-processing approach where they immersed the printed structures in solutions with varying concentrations of sodium bicarbonate (1, 3, and 5% on a weight basis). As shown in Figure 5c, time-dependent changes in the color value and intensity (from yellow to red) were observed. The observed effects mark the transition of the curcumin from a keto conformational state (fluorescent yellow) to its enol conformational state (bright red). Moreover, the authors comprehensively elucidated the resultant color changes using UV absorbance.

Combining the effects of external and internal pH stimuli, Ghazal et al. studied the color change effects of printed structures through mass diffusion in different food models [53]. The authors used an anthocyanin- and starch-based material system composed of red cabbage and different concentrations of potato starch. First, as shown in Figure 5a, the color change effect was studied for different heights of printed single-material structures through the spraying of variable pH solutions. The printed structures were uniformly sprayed twice using 0.75 mL of the solutions (pH 2.5 and 8) and the color change behavior was observed directly after printing and after 1.5 h. As shown in Figure 5a, the color change behavior was dependent on the thickness of the printed structures due to diffusion, which is time-dependent. For all printed thicknesses (6, 8, and 10 mm), it was observed for the pH 8 solution at the vertical section that the structure’s core was darker than the perimeter after 1.5 h, indicating that the sprayed solution did not diffuse in the required time to the core. Second, Ghazal et al. studied the influence of the diffusion boundary’s geometry and the thickness of the printed structures in printed bi-material composites [53]. The investigation was performed using three models, shown in Figure 5b, using the previous material combined with an acidic material phase based on lemon and potato starch. The acidic phase is located at the bottom of Models 1 and 2 and located in the middle of Model 3. The material phases were printed at different thicknesses of 6 and 4 mm, for Models 1 and 2 respectively. The results showed that Models 2 and 3 reached an equilibrium (in color change) due to diffusion after approximately 1.5 h, while Model 1 reached an equilibrium after 3 h. Compared to Model 2, the response time of Model 1 was lower due to the higher thickness. In addition, the diffusion boundary of Model 3 had the highest surface, leading to an increased diffusion rate.

**Figure 6:**
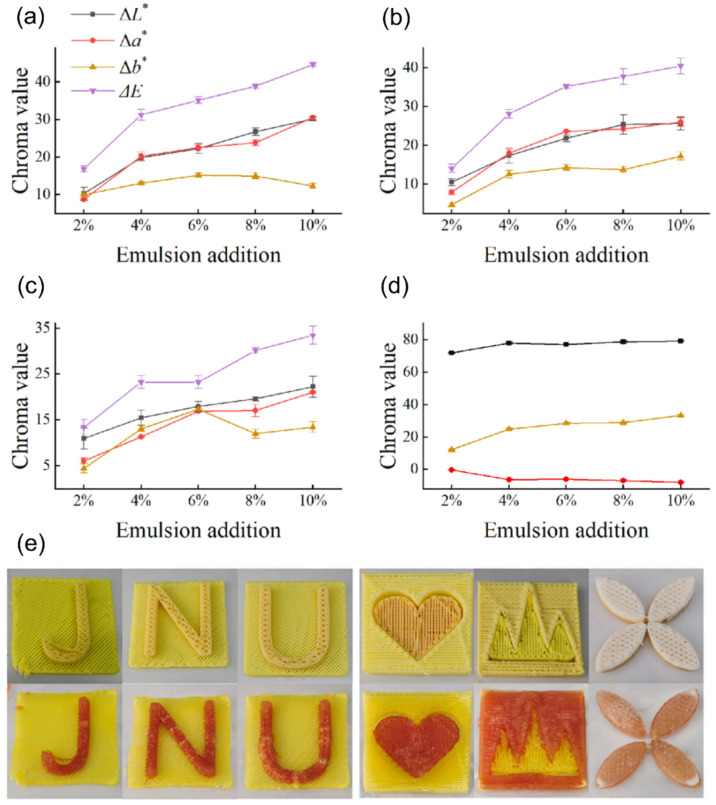
Effects of curcumin-based emulsion concentration and microwave stimulation on the chroma value of a lotus root material system (reproduced and adapted from [55] with permission from Elsevier, 2021): (**a**) chroma value after 1 min of microwave stimulation; (**b**) chroma value after 2 min of microwave stimulation; (**c**) chroma value after 3 min of microwave stimulation; (**d**) native material without microwave stimulation; (**e**) printed multi-material food composites.

Another post-processing or external stimulation for color change is through dehydration. From a consumption perspective, this external stimulation method presents a more feasible approach to color change (compared to external pH stimulation), as inducing thermal transitions is imperative for most 3D-printed food materials. Chen et al. investigated the effect of microwave dehydration (MD) on the color change kinetics of curcumin-based 3D-printed structures [55]. In this study, the authors studied the color change with respect to the dehydration time of printed structures by the addition of curcumin emulsion and NaHCO_3_ (pH = 8.15) to a single-phase lotus root material. As shown in Figure 6, the colorimetry of the printed samples was characterized at different dehydration times of 1–3 min at a constant power level of 280 W. Using microwave dehydration, the NaHCO_3_ was decomposed into Na_2_CO_3_, which enhanced the alkalinity of the material. In addition, the authors observed (through confocal laser microscopy) that the emulsion particles aggregate during the application of MD. Thus, they inferred that some emulsion particles burst, which led to the exposure of the curcumin particulates to alkaline conditions, resulting in the deprotonation of the curcumin, denoted by the yellow to red color shift, as shown in Figure 6e.

### 3.2. Shape Morphing and Dynamic Textures

Textures of foods and food foams are defined as mechanical, structural, and surface properties [60]. Dynamic textures denote the time-dependent changes in the mechanical, structural, or surface properties of printed structures, causing a controlled change in the textural properties. Such dynamic textures of 3D-printed food structures can be achieved using triggered shape-morphing effects. Shape morphing denotes structural deformation causing the transition of structures between two or more different morphological states. To illustrate, defined stimuli-driven stresses are developed in the structures, and due to dimensional or material mismatch, such stresses induce specific deformations, such as bending, torsion, distortion, linear and non-linear expansion/contraction, and surface curl. Controlled deformation in the printing of food materials and structures has progressed rapidly in the last few years, as the fundamental understanding of shape change kinetics was already established for synthetic organic and inorganic materials [30,61]. In order to not trivialize the scientific progress, the published investigations remain a largely substantial scientific step when considering the challenges in geometrical designs, multi-material matching/mismatching, and tailoring of food material responses towards external stimuli. To emphasize this, the structuring of shape-morphing food assemblies is a direct adaptation of 4D printing in engineering applications, which focuses on the processing of stimuli-responsive smart materials and structures. In engineering applications, the processing of smart materials or structures is performed to achieve specific functionalities typically in sensing and actuation [29]. Compared to shape-morphing foods, a broader range of materials is used in engineering applications, including monolithic and composite material approaches (Ahmed et al., 2021; Pinho et al., 2020). With this in mind, the available literature on the 4D printing of shape-morphing food structures still encompasses a wide range of food materials, including hydrogels and fiber-, starch-, fruit-, and animal-based material systems [42,45,62].

The kinetics of shape-morphing foods are generally engineered using one or a combination of the following structure design approaches:

(1) Bi- or multi-material systems;
(2) Design configuration;
(3) Active–passive layers.

For the different structure design approaches, the dynamics of the shape-morphing process are driven by relative expansion or contraction between either different layers (materials) or repetitive/non-repetitive design patterns during hydration or dehydration. Considering hydration, the rate and capacity of water absorption control the swelling anisotropy, which causes a non-uniform distribution of stresses within the structure [63]. Such non-uniformity in the development of internal stresses is the driving force for the material transformation from one configuration to another. In dehydration-triggered stimulations, structural deformation occurs due to the relative contraction between different materials or design configurations according to their own thermal and mass transfer properties. Thus, it is mainly controlled using moisture loss through the thermal and diffusion properties of food formulations. However, mass diffusion (hydration and dehydration) is not the only approach to controlling or inhibiting shape-morphing kinetics, it can also be controlled through thermal expansion (for example, cellular structures), molecular transformations (for example, starch gelatinization), or organic growth in active-passive structures [30,64].

**Figure 7:**
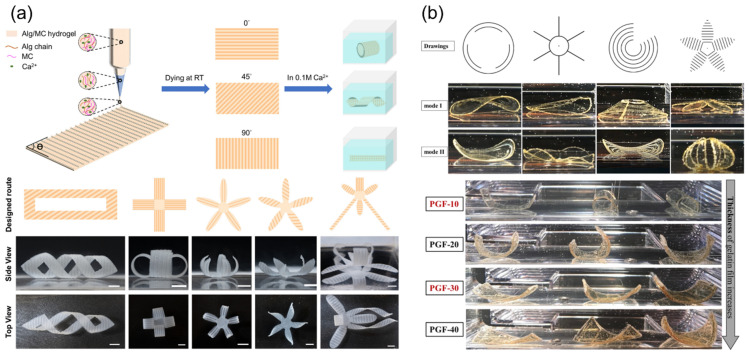
Hydration-triggered shape-morphing multi-material structures: (**a**) illustration and pictures of shape-morphing configurations using 3D-printed Alg/MC hydrogels (reproduced from [39] under the terms of Creative Commons CC-BY license); (**b**) effect of 3D-printed EC pattern and gelatin film thickness on shape-morphing behavior of patterned structures (reproduced and adapted from [72] with permission from Elsevier, 2022).

Currently, the available literature on the 4D printing of shape-morphing food structures encompasses a range of imperative research directions, such as investigating the design-driven deformation effects in multi-material structures, the deformation kinetics by using different drying mechanisms, and the effects of fats, salts, and other additives (variable thermal and diffusion properties) on deformation kinetics [39,65,66,67,68,69,70,71]. Scientific studies presented by Pulatsu et al. and Lai et al. investigated the swelling and shape-morphing behaviors of hydration-triggered 4D-printed edible food composites [39,72]. As shown in Figure 7a, the study by Lai et al. showed the design-controlled morphing of printed alginate/methylcellulose (Alg/MC) hydrogel structures. The shape-morphing effects were engineered by inducing a network density gradient across the printing plane. To illustrate, the density gradient resulted in a non-uniform swelling behavior, leading to relative expansion across the printing plane and the transformation of 3D-printed flat structures from one configuration to another, forming complex 3D morphologies. Moreover, the pre-defined deformations were triggered by the immersion of the printed flat sheets in calcium chloride solutions (CaCl_2_). In this study, the authors introduced a novel approach for inducing such hydration-triggered transformations using network gradient discrepancies. This was achieved by printing three-layered sheets, the first layer of which was a uniform film. The second and third layers were printed as a strip pattern uniformly interspaced at different angles. Furthermore, to create the network density gradient, the authors dried the printed structures at room temperature to create a compressed network and dense regions of the three-layered areas, while the single-layer areas produced lower network densities. Using a different approach to induce hydration-triggered transformations in food structures, the study presented by Pulatsu et al. investigated shape-morphing effects and configurations using multi-materials or composite systems by the 3D printing of ethyl cellulose (EC) patterns on pure gelatin and glycerol–gelatin films, as shown in Figure 7b. The presented study showed a novel approach for achieving shape-morphing effects using swelling anisotropy driven by the hydrophilic behavior of gelatin in relation to the hydrophobicity of EC. The authors investigated the effects of film thickness, aspect ratio, swelling properties, and printing patterns on the shape-morphing properties when subjecting the films to water as a stimulus. One of the most important metrics the authors used to quantify the dependencies of the shape-morphing properties was the response capacity of the patterned structures [72]. In the study, the response capacity was considered to be high when flat structures reached their maximum deformation in a shorter period of time in response to the applied hydration stimulus. Regarding the dependency of the response capacity on the film thickness, as shown in Figure 7b, the authors observed an inverse relationship between the film thickness and the final curvature of the structures. To induce simple bending, the authors printed parallel EC strips on gelatin films. Upon hydration of the patterned films, curvatures (defined as the reciprocal of a circle’s radius) ranging from 0.045 to 0.576 mm^−1^ were obtained, depending of the film’s thickness. The authors presented promising shape-morphing effects using different distributions of the EC on the gelatin film, which proved to be an imperative method for elucidating hydration-dependent shape-morphing kinetics. However, in this study, 3D printing was only used for the deposition of EC patterns on pre-prepared gelatin films. Therefore, the application of dual extrusion can be a step forward for the processing of hydration-triggered morphing food structures.

**Figure 8:**
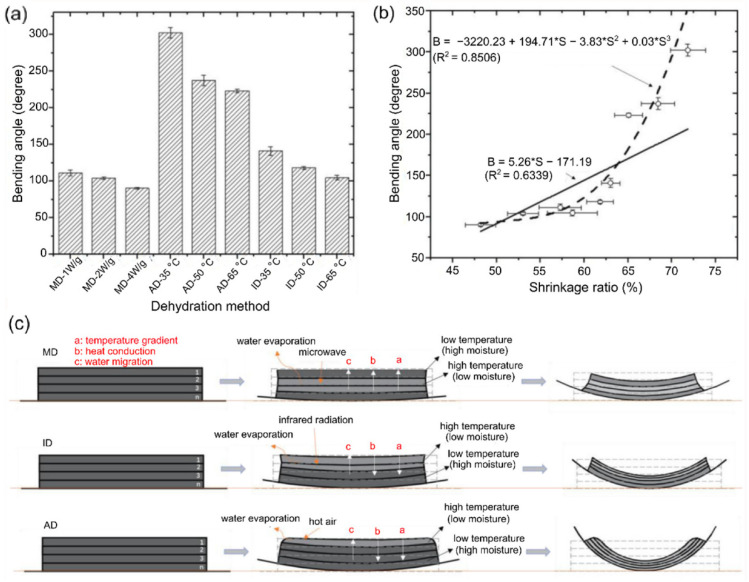
Effects of dehydration temperature, power, and method on the shrinking and bending behavior of 3D-printed active starch-based layer on a passive plastic film (reproduced and adapted from [73] with permission from Elsevier, 2021): (**a**) resultant bending angle with respect to variable air drying (AD) and infrared drying (ID) temperatures and variable microwave drying (MD) powers; (**b**) correlation between the shrinkage ratio and the bending angle; (**c**) schematic representation of the heating and bending kinetics driven by different dehydration methods (MD, ID, and AD).

Considering industrial applications, namely in pasta thermal treatment or cooking, hydration-triggered geometrical change is a relatively promising 4D printing application for its prospects in the reduction of packaging, storage space, and transportation costs. However, the number of studies focusing on hydration-triggered structures is still limited as they require the use of complex multi-material hydrogel systems where the printability is lower and due to the viscoelastic-induced geometrical inaccuracies (namely a high surface roughness) in printed hydrogel structures [39]. On the other hand, dehydrated-triggered shape-morphing kinetics and geometrical transformations are the most studied fields, as deformations are mainly controlled using mass transfer, which can be applied to a wider range of food material systems. Studying the influence of different heating methods on shape-morphing behavior, Liu et al. compared air drying (AD), infrared drying (ID), and microwave drying (MD) using the simple bending of an active food material system printed onto a thin passive layer (0.2 mm) of food-grade PA/PE film [73]. In the study, the authors set the heating temperatures for the AD and ID at 35, 50, and 65 °C while they used MD power of 1.0, 2.0, and 4.0 W/g. The investigation was performed using a starch-based material composed of potato flakes, potato starch, fructose syrup, and xanthan gum. Structures were printed in a bilayer approach, where one active layer was printed to a height of 1.2 mm. The geometrical design choice of the authors reflects the insignificant need for complex geometrical designs, which complicates the elucidation of the shape-morphing behavior in relation to the used drying methods. As shown in Figure 8a, the bending angle was observed to be negatively correlated with both the heating temperatures (for AD and ID) and the microwave power (MD). Specifically, the bending angle decreased from 111.30 to 90.29 °C during the application of MD from 1 W/g to 4 W/g, respectively. The authors observed that the deformation speed decreased with respect to the microwave power, which is indicative of reaching a plateau region at a certain temperature where no subsequent dehydration occurred. Therefore, increasing the microwave power resulted in a rapid volume shrinkage where the rate of mass transfer decreased, leading to a decreased shape transformation speed. A similar effect was observed for the experiments performed using AD and ID. Liu et al. observed that the application of traditional AD led to higher bending angles compared to the other methods, as shown in Figure 8a. The authors attributed this effect to what they defined as an accumulation bending effect, which is caused by moving the water evaporation boundary from the external layer to the internal layers [74]. As illustrated in Figure 8c, during AD, the heat was first absorbed by the external layer, which caused water evaporation and initiated the contraction and bending. Then, the water evaporation and contraction propagate to the internal layers. However, during the MD, the evaporation boundary propagated from the internal layers towards the external layers. The inverse propagation of the moving boundary caused inhibition of the bending as the external layers restricted the contraction of the internal layers. Accordingly, the investigations presented by Lui et al. are imperative to the understanding of the kinetics of different heating methods applied in dehydration-triggered deformations.

**Figure 9:**
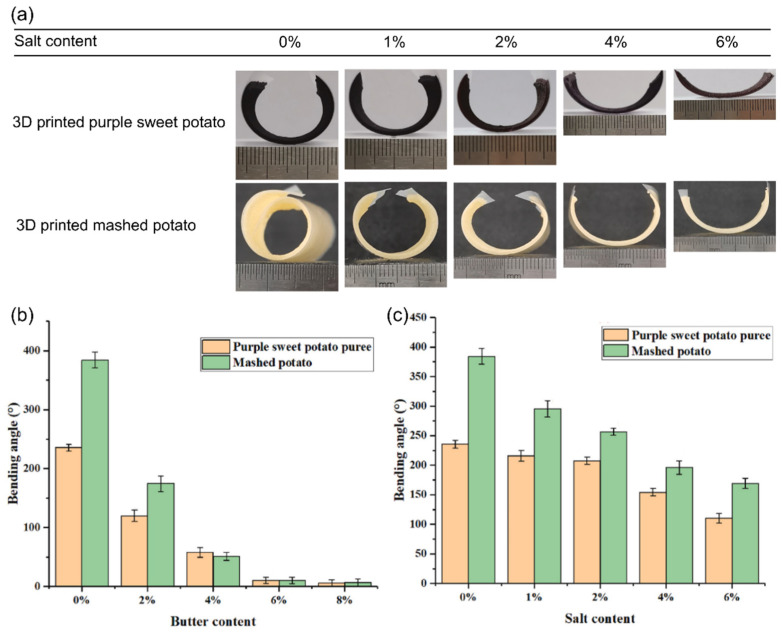
Bending behavior of 3D-printed active mashed potato and purple sweet potato layers with respect to salt and butter contents using MD (reproduced and adapted from [67] with permission from Elsevier, 2021): (**a**) images of active-passive layer bending at different salt (sodium chloride) concentrations; (**b**) influence of butter content on the bending angle; (**c**) influence of salt content on the bending angle.

Another important research objective in the shape morphing of food structures is elucidating the effects of fats, salts, and additives addition during dehydration-triggered deformation. The investigations related to salt and butter incorporation (for the manipulation of thermal and diffusion properties) present a new perspective on understanding the relationship between the dielectric properties of foods and shape morphing during dehydration approaches using electromagnetic fields. In addition, such a direction of research is relevant for the controlled tailoring of food material systems applied in shape-morphing applications, which can be further extended for combining sensory modulation with textural transformations. A study by C. He, M. Zhang, and S. Devahastin (2021) investigated the effects of edible salt and butter contents on the dielectric properties and interrelated bending angles of the shape morphing of starch-based material systems [67]. Similar to the study highlighted earlier by Liu et al., the experiment adopted a directional deformation approach using the simple bending of one active printed layer on a thin food-grade PET/PE sheet. The investigations were performed using two food material systems using purple sweet potato pure (PSPP) and mashed potato (MP) with a salt addition of 1% to 6% *w*/*v* or with a butter addition of 2% to 8% *w*/*v*. For the application of the stimulation in the form of dehydration, a 2.0 W/g microwave dehydration process was used. While the moisture content was kept at a constant level for both material systems (including all salt and butter concentrations), as shown in Figure 9a, the deformation of the printed structures decreased with the increase in both the salt and butter contents for both material systems. The addition of salt in a food material system affects the dielectric properties, inducing changes in the penetration of microwaves and the heating rate, thereby affecting the dehydration process [75]. The analysis of the dielectric properties presented in the study showed a significant increase in the dielectric loss factor of the PSPP and MP by 190.3% and 167.5%, respectively, when the salt content was increased from 0 to 6%. As a result, as shown in Figure 9b, the bending angle of the PSPP and MP structures decreased from 235.5° to 110.2° and from 384.4° to 169.4°, respectively. To illustrate, the increase in the dielectric loss factor increased the material’s conductivity, leading to higher heating rates. Subsequently, this resulted in more extensive moisture loss and hardening on the structure’s surface, thus inhibiting the deformation of the printed structures. Most importantly, the discrepancy between the resultant bending angles of the PSPP and MP systems, shown in Figure 9b,c, are indicative of the role of the material composition in the development of internal stress during mass transfer. A more extensive reduction in the bending angles can be observed in both material systems with the increase in the butter content. However, the authors reported a significant reduction in the dielectric loss factor at the same time. The authors explained the large reduction in the bending angle as due to the loss in adhesion in the interface between the active and passive layers, which was caused by phase separation during the melting of fats.

**Figure 10:**
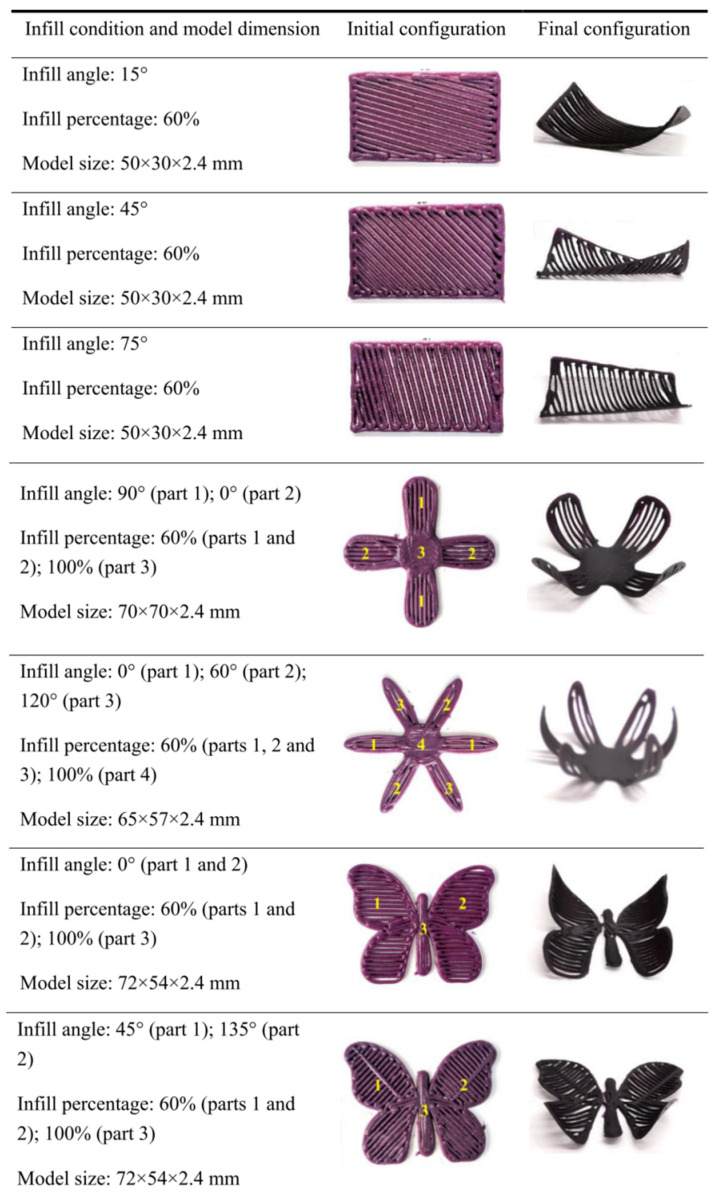
Dehydration-triggered shape morphing of different design configurations as influenced by the internal structure (reproduced from [65] with permission from American Chemical Society, 2020).

By processing food structures using 3D printing, the design of solid structures usually includes a shell (perimeter) and an infill (internal structure). If both parts are considered as different material phases, the internal structure has a higher relative density at high infill levels. Correspondingly, the developed stresses in the internal structure during dehydration are the main driving force for shape morphing. Therefore, through the manipulation of the internal structure design, the control of shape-morphing effects can be a simpler applied approach compared to the use of multi-material structures. As an example, C. He, M. Zhang, and S. Devahastin (2020) influenced shape-morphing deformation patterns by manipulating the infill parameters [65]. In addition, the study investigated the bending of structures in relation to microwave dehydration time. Similar to the study mentioned before [67], in their latter study, the same authors used a starch-based material system based on purple sweet potato. As shown in Figure 10, the authors controlled the deformation only by manipulating the internal path and the infill densities. By printing simple single-layer sheets, a bending or torsion-induced deformation occurred perpendicularly to the direction of the internal structure’s alignment. However, the directionality of the deformation was highly dependent on the configuration of the internal structure. Finally, further investigations of such an approach for controlling shape-morphing behavior using complex internal structure configurations are imperative for the realization of more complex deformation-driven food structures.

### 3.3. Cell Culturing for Texture and Nutritional Development

As mentioned in Section 2, the application of thermal stabilization to achieve textural, flavor, and nutritional development cannot be considered 4D printing, as most highly consumed staple foods (in the application of 3D printing or traditional processing) containing starches and proteins are traditionally submitted to heat treatment and dehydration to induce essential physiochemical changes for human consumption and storage. However, according to the stated definition of 4D food printing, time-dependent changes in texture, flavor, and nutrition can be achieved through the culturing of plant- or animal-based cells in structured scaffolds. The studies that have focused on the application of 3D printing in cell culturing have not typically considered this approach as 4D printing. Nonetheless, the consideration of this approach as 4D printing can be attributed to the fact that structures are printed with additional functionality that develops and progresses over time through biological growth, which does not occur in typical 3D-printed food structures.

**Figure 11:**
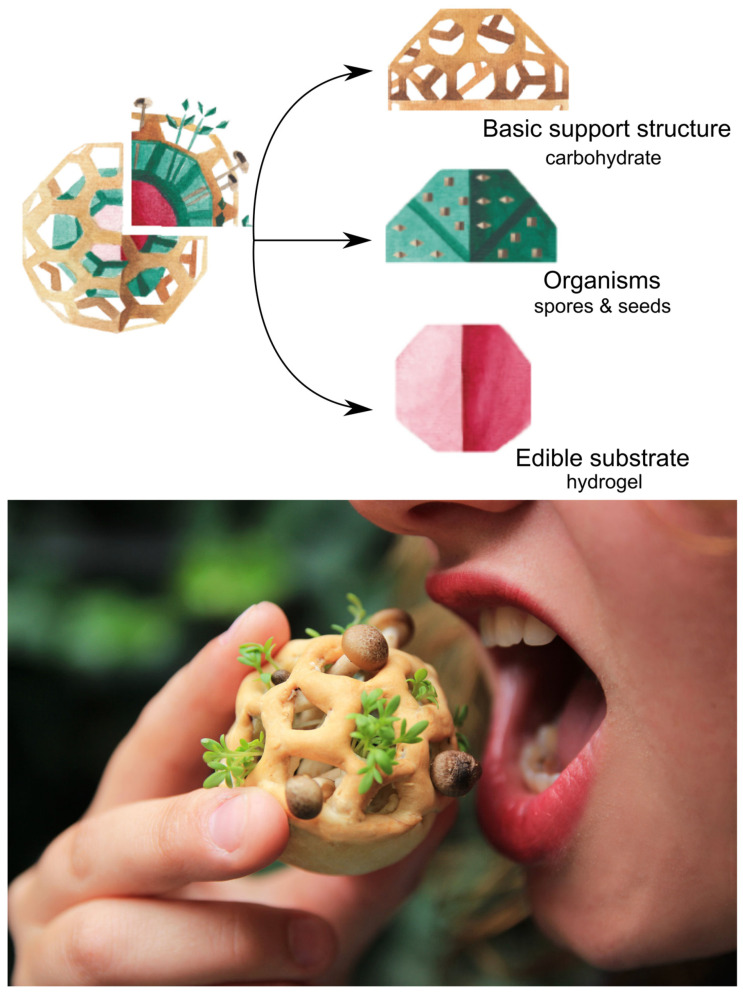
Illustration and picture of edible food growth consisting of a hydrogel substrate incorporating spores and seeds encased in a 3D-printed starch-based support structure (reproduced from [77]).

In general terms, cell culturing in printing applications (namely medical and food applications) is associated with the printing of scaffold structures with plant- or animal-based cells incorporated into a medium that promotes cellular growth and a scaffold design that supports proliferation [76]. Prior to the 4D printing of scaffolds using plant- or animal-based cells containing material systems for food applications, two other approaches were explored using 3D printing. The first approach was not a scientific investigation but rather a food design approach performed using 3D printing, which was implemented by a food designer in collaboration with the University of Eindhoven and TNO in 2014 [77]. The project “Edible Food Growth” introduced 3D-printed food assemblies or composites supporting various biological growth, as shown in Figure 11. In this approach, spore-forming organisms and seeds were incorporated into a hydrogel-based edible substrate. Then, the edible substrate was incorporated into a carbohydrate-based 3D-printed support structure, allowing for time-dependent organic growth within the assembly. This concept or approach presents a novel avenue where the texture, flavor, and nutrition of food structures are time- or growth-dependent, delivering to the consumers a dynamic sensory experience and nutritional facts. However, according to the stated definition in Section 2, this approach cannot be considered as 4D food printing as the structures are not exclusively processed using a 3D printing approach. Still, adopting such an approach in a systematic scientific investigation is imperative for the elucidation of textural and flavor changes over time with respect to the incorporated cultures, support structure, and growth substrate.

The second investigated approach, serving as a foundation for 4D printing in the form of plant-cell culturing, was the encapsulation of plant cells in a model food material system [22]. Vancauwenberghe et al. investigated the possibility of textural modulation and cell viability of low-methoxylated pectin gels incorporating isolated lettuce leaf cells (*Valerianella locusta*, L. var. ‘Gala’). In the study, the authors investigated the effects of cell encapsulation on the material-process interaction and printability of the cell-containing gel structures. The results show that structures with up to 5 × 10^6^ cells/mL were successfully printable. Regarding the material and cell-concentration-dependent mechanical properties of the printed structures, the mechanical strength increased with the pectin concentration, which was expected due to the formation of a stronger network at higher concentrations. On the other hand, the mechanical strength decreased, indicating a structural softening, with the increase in the cell concentration. In addition, the study investigated the cell viability depending on the pectin concentration. However, the authors did not investigate the time-dependent growth of the cell culture. Therefore, according to the definition stated in Section 2, the mentioned study can be considered as 3D printing of nutritionally enriched pectin-based gels and not 4D food printing.

**Figure 12:**
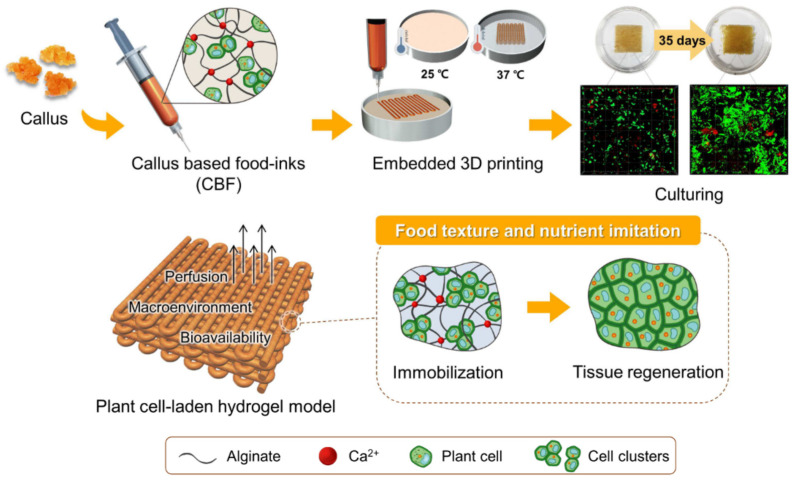
Schematic illustration of plant-based cell culturing using callus-based food material system and embedded 3D printing of plant cell-laden hydrogel scaffolds (reproduced from [23] with permission from Elsevier, 2020).

**Figure 13:**
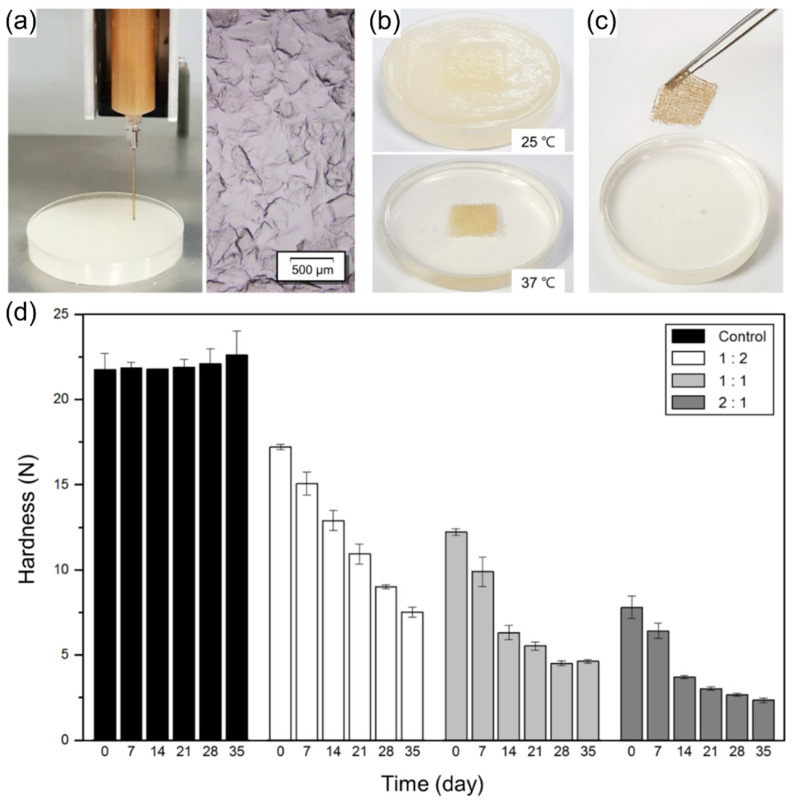
Embedded printing process and hardness of the plant cell-laden hydrogel scaffolds (reproduced and adapted from [23] with permission from Elsevier, 2020): (**a**) material deposition in a gelatin gel containing CaCl_2_; (**b**) destabilization of the gelatin gel at 37 °C; (**c**) removal of the printed scaffold for further incubation; (**d**) hardness levels at different culturing times for different rations of callus dispersion and alginate.

As an example of plant-based cell culturing using 4D printing, Park, Kim, and Park (2020) established a novel approach for cell culturing using model material scaffolds in food structures [23]. The study applied a 3D printing approach for the implementation of a plant cell-laden hydrogel model for achieving time-dependent textural changes. As shown in Figure 12, the authors performed a study using callus-based food inks composed of separated carrot callus cells embedded in an alginate matrix. With the purpose of producing variable edible cellular tissues, the authors investigated the correlation between printability, proliferation efficiency, and time-dependent textural properties of the printed structures, and elucidated the properties at different initial cell densities of the callus-based materials. In this study, the callus-based materials were prepared by blending a callus dispersion with 4% alginate at ratios of 1:2, 1:1, and 2:1 (*w*/*w*). Moreover, the authors used an embedded 3D printing approach to suspend the printed scaffolds in a gelatin gel containing CaCl_2_ for crosslinking of the alginate. Through optical density and confocal microscopy quantification, the printed plant cell-laden scaffolds with different callus contents showed high viability and prolonged cell growth over a 35-day culturing period. To quantify the time- and cell proliferation-dependent textural changes, the authors performed compression tests at 7-day intervals. The results showed that, for the non-cell containing alginate gel, there were no significant differences in the hardness with respect to time, as shown in Figure 13. This indicates that the modulation of the hardness levels was dependent on the cell growth. For the different initial cell gel concentrations, a decrease in hardness was observed. Such a decrease in hardness was explained by the matrix inhomogeneity caused by the increase in the non-uniformity of cell distribution during culturing. Furthermore, the hardness was reduced to 50% of the initial level during culturing, which occurred on day 28 for the 1:2 gel, on day 21 for the 1:1 gel, and on day 14 for the 2:1 gel. However, as an important statement, the authors recommended further investigation of the cell aggregation between immobilized cell clusters for the possible improvement of the mechanical strength of the printed structures. The study did not investigate the full time-dependent sensory changes of such structures; however, it can be stated that the 4D printing cell culturing approach can be further studied and used for the time-dependent modulation of sensory perception and nutrition.

## 4. Conclusions and Future Considerations in 4D Food Printing

As a direct extension of 3D printing, 4D food printing presents a new approach to the designing and processing of dynamic foods. The term dynamic denotes the time-dependent dimension of 3D-printed foods, which enables the transformation of structures from one configuration to another. Currently, the research is focused on three main categories, which are stimulated color change, stimulated shape morphing, and cell culturing. As illustrated in Figure 2, the different time-dependent behaviors, such as color and shape changes, are stimulated through various approaches. The processing of time-dependent food structures enables the interaction and consumption of foods that offer new sensory perceptions. For instance, dehydration-triggered shape morphing and cell culturing enable textural variation depending on the post-processing degree and incubation time, respectively. However, since 4D food printing is still an emerging processing approach, further comprehensive investigations and developments are required to precisely modulate and control the targeted functionalities. Furthermore, through the mentioned studies, it can be directly inferred that the application of food materials in 4D printing needs to be extended, as investigations are still limited to hydrogels and starch-based materials. Considering the stimulated color changes, the understanding of the material- and boundary-dependent diffusion is imperative for the processing of structures with controlled color change behavior. The use of multi-material deposition and in-line dehydration can enable the creation of complex shape-morphing configurations while eliminating post-processing. Most importantly, 4D transformations have the potential to be utilized to elucidate diffusive effects as well as the effect of physiochemical transformations in relation to morphological changes in bulk food structures. Moreover, the degree of shape morphing can be controlled through in-line monitoring. Finally, the technological adaption of these processing approaches can further enhance the application of 4D food printing.

## Data Availability

The data used to support the findings of this study can be made available by the corresponding author upon request.

## References

[1] Sun J., Peng Z., Zhou W., Fuh J.Y.H., Hong G.S., Chiu A. (2015). A Review on 3D Printing for Customized Food Fabrication. Proceedings of the Procedia Manufacturing.

[2] Chen Y., Zhang M., Sun Y., Phuhongsung P. (2022). Improving 3D/4D Printing Characteristics of Natural Food Gels by Novel Additives: A Review. Food Hydrocoll..

[3] Zhao L., Zhang M., Chitrakar B., Adhikari B. (2021). Recent Advances in Functional 3D Printing of Foods: A Review of Functions of Ingredients and Internal Structures. Crit. Rev. Food Sci. Nutr..

[4] Derossi A., Caporizzi R., Ricci I., Severini C. (2018). Critical Variables in 3D Food Printing. Fundamentals of 3D Food Printing and Applications.

[5] Godoi F.C., Prakash S., Bhandari B.R. (2016). 3d Printing Technologies Applied for Food Design: Status and Prospects. J. Food Eng..

[6] Severini C., Azzollini D., Albenzio M., Derossi A. (2018). On Printability, Quality and Nutritional Properties of 3D Printed Cereal Based Snacks Enriched with Edible Insects. Food Res. Int..

[7] Fahmy A.R., Becker T., Jekle M. (2020). 3D Printing and Additive Manufacturing of Cereal-Based Materials: Quality Analysis of Starch-Based Systems Using a Camera-Based Morphological Approach. Innov. Food Sci. Emerg. Technol..

[8] Severini C., Derossi A., Azzollini D. (2016). Variables Affecting the Printability of Foods: Preliminary Tests on Cereal-Based Products. Innov. Food Sci. Emerg. Technol..

[9] Liu Z., Bhandari B., Prakash S., Mantihal S., Zhang M. (2019). Linking Rheology and Printability of a Multicomponent Gel System of Carrageenan-Xanthan-Starch in Extrusion Based Additive Manufacturing. Food Hydrocoll..

[10] Kim H.W., Bae H., Park H.J. (2017). Classification of the Printability of Selected Food for 3D Printing: Development of an Assessment Method Using Hydrocolloids as Reference Material. J. Food Eng..

[11] Holland S., Tuck C., Foster T. (2018). Selective Recrystallization of Cellulose Composite Powders and Microstructure Creation through 3D Binder Jetting. Carbohydr. Polym..

[12] Jonkers N., van Dommelen J.A.W., Geers M.G.D. (2020). Experimental Characterization and Modeling of the Mechanical Behavior of Brittle 3D Printed Food. J. Food Eng..

[13] Zhang M., Liu Z., Bhandari B., Wang Y. (2017). 3D Printing: Printing Precision and Application in the Food Sector. Trends Food Sci. Technol..

[14] Fahmy A.R., Amann L.S., Dunkel A., Frank O., Dawid C., Hofmann T., Becker T., Jekle M. (2021). Sensory Design in Food 3D Printing—Structuring, Texture Modulation, Taste Localization, and Thermal Stabilization. Innov. Food Sci. Emerg. Technol..

[15] Maniglia B.C., Fahmy A.R., Jekle M., Le-Bail P., Le-Bail A., Sandhu K., Singh S. (2022). Three-Dimensional (3D) Food Printing Based on Starch-Based Inks: Crucial Factors for Printing Precision. Food Printing: 3D Printing in Food Industry.

[16] Vancauwenberghe V., Delele M.A., Vanbiervliet J., Aregawi W., Verboven P., Lammertyn J., Nicolai B. (2018). Model-Based Design and Validation of Food Texture of 3D Printed Pectin-Based Food Simulants. J. Food Eng..

[17] Ko H.J., Wen Y., Choi J.H., Park B.R., Kim H.W., Park H.J. (2021). Meat Analog Production through Artificial Muscle Fiber Insertion Using Coaxial Nozzle-Assisted Three-Dimensional Food Printing. Food Hydrocoll..

[18] Escalante-Aburto A., Trujillo-de Santiago G., Álvarez M.M., Chuck-Hernández C. (2021). Advances and Prospective Applications of 3D Food Printing for Health Improvement and Personalized Nutrition. Compr. Rev. Food Sci. Food Saf..

[19] Fahmy A.R., Jekle M., Becker T. (2022). Texture Modulation of Starch-Based Closed-Cell Foams Using 3D Printing: Deformation Behavior beyond the Elastic Regime. J. Texture Stud..

[20] Fahmy A.R., Vogt U.T., Jekle M., Becker T. (2022). Hardness Targeted Design and Modulation of Food Textures in the Elastic-Regime Using 3D Printing of Closed-Cell Foams in Point Lattice Systems. J. Food Eng..

[21] Pereira T., Barroso S., Gil M.M. (2021). Food Texture Design by 3d Printing: A Review. Foods.

[22] Vancauwenberghe V., Baiye Mfortaw Mbong V., Vanstreels E., Verboven P., Lammertyn J., Nicolai B. (2019). 3D Printing of Plant Tissue for Innovative Food Manufacturing: Encapsulation of Alive Plant Cells into Pectin Based Bio-Ink. J. Food Eng..

[23] Park S.M., Kim H.W., Park H.J. (2020). Callus-Based 3D Printing for Food Exemplified with Carrot Tissues and Its Potential for Innovative Food Production. J. Food Eng..

[24] Shahbazi M., Jäger H., Ettelaie R. (2021). Application of Pickering Emulsions in 3D Printing of Personalized Nutrition. Part II: Functional Properties of Reduced-Fat 3D Printed Cheese Analogues. Colloids Surf. A Physicochem. Eng. Asp..

[25] Teng X., Zhang M., Mujumdar A.S. (2021). 4D Printing: Recent Advances and Proposals in the Food Sector. Trends Food Sci. Technol..

[26] Chen J., Teng X., Zhang M., Bhandari B., Adhikari B., Yu D. (2023). 5D Food Printing with Color Change Induced by Probiotic Growth in a Starch-Protein-Based Gel System. Food Bioprocess. Technol..

[27] Pinho A.C., Buga C.S., Piedade A.P. (2020). The Chemistry behind 4D Printing. Appl. Mater. Today.

[28] Ahmed A., Arya S., Gupta V., Furukawa H., Khosla A. (2021). 4D Printing: Fundamentals, Materials, Applications and Challenges. Polymer.

[29] Mallakpour S., Tabesh F., Hussain C.M. (2021). 3D and 4D Printing: From Innovation to Evolution. Adv. Colloid Interface Sci..

[30] Momeni F., Ni J. (2020). Laws of 4D Printing. Engineering.

[31] Le Duigou A., Correa D., Ueda M., Matsuzaki R., Castro M. (2020). A Review of 3D and 4D Printing of Natural Fibre Biocomposites. Mater. Des..

[32] Alshebly Y.S., Nafea M., Mohamed Ali M.S., Almurib H.A.F. (2021). Review on Recent Advances in 4D Printing of Shape Memory Polymers. Eur. Polym. J..

[33] Demoly F., Dunn M.L., Wood K.L., Qi H.J., André J.C. (2021). The Status, Barriers, Challenges, and Future in Design for 4D Printing. Mater. Des..

[34] Haleem A., Javaid M., Singh R.P., Suman R. (2021). Significant Roles of 4D Printing Using Smart Materials in the Field of Manufacturing. Adv. Ind. Eng. Polym. Res..

[35] Sydney Gladman A., Matsumoto E.A., Nuzzo R.G., Mahadevan L., Lewis J.A. (2016). Biomimetic 4D Printing. Nat. Mater..

[36] Raviv D., Zhao W., McKnelly C., Papadopoulou A., Kadambi A., Shi B., Hirsch S., Dikovsky D., Zyracki M., Olguin C. (2014). Active Printed Materials for Complex Self-Evolving Deformations. Sci. Rep..

[37] Zhang Q., Zhang K., Hu G. (2016). Smart Three-Dimensional Lightweight Structure Triggered from a Thin Composite Sheet via 3D Printing Technique. Sci. Rep..

[38] Mulakkal M.C., Trask R.S., Ting V.P., Seddon A.M. (2018). Responsive Cellulose-Hydrogel Composite Ink for 4D Printing. Mater. Des..

[39] Lai J., Ye X., Liu J., Wang C., Li J., Wang X., Ma M., Wang M. (2021). 4D Printing of Highly Printable and Shape Morphing Hydrogels Composed of Alginate and Methylcellulose. Mater. Des..

[40] Ding Z., Yuan C., Peng X., Wang T., Qi H.J., Dunn M.L. (2017). Direct 4D Printing via Active Composite Materials. Sci. Adv..

[41] Kotikian A., Truby R.L., Boley J.W., White T.J., Lewis J.A. (2018). 3D Printing of Liquid Crystal Elastomeric Actuators with Spatially Programed Nematic Order. Adv. Mater..

[42] Ghazal A.F., Zhang M., Mujumdar A.S., Ghamry M. (2022). Progress in 4D/5D/6D Printing of Foods: Applications and R&D Opportunities. Crit. Rev. Food Sci. Nutr..

[43] Phuhongsung P., Zhang M., Bhandari B. (2020). 4D Printing of Products Based on Soy Protein Isolate via Microwave Heating for Flavor Development. Food Res. Int..

[44] Wang W., Yao L., Zhang T., Cheng C.Y., Levine D., Ishii H. (2017). Transformative Appetite: Shape-Changing Food Transforms from 2D to 3D by Water Interaction through Cooking. Proceedings of the 2017 CHI Conference on Human Factors in Computing Systems.

[45] Navaf M., Sunooj K.V., Aaliya B., Akhila P.P., Sudheesh C., Mir S.A., George J. (2022). 4D Printing: A New Approach for Food Printing; Effect of Various Stimuli on 4D Printed Food Properties. A Comprehensive Review. Appl. Food Res..

[46] Tsaplev Y.B., Lapina V.A., Trofimov A.V. (2020). Curcumin in Dimethyl Sulfoxide: Stability, Spectral, Luminescent and Acid-Base Properties. Dye Pigment..

[47] Tena N., Asuero A.G. (2022). Up-To-Date Analysis of the Extraction Methods for Anthocyanins: Principles of the Techniques, Optimization, Technical Progress, and Industrial Application. Antioxidants.

[48] Rodriguez-Saona L.E., Wrolstad R.E. (2001). Extraction, Isolation, and Purification of Anthocyanins. Curr. Protoc. Food Anal. Chem..

[49] Phuhongsung P., Zhang M., Devahastin S. (2020). Influence of Surface PH on Color, Texture and Flavor of 3D Printed Composite Mixture of Soy Protein Isolate, Pumpkin, and Beetroot. Food Bioprocess Technol..

[50] Spence C. (2019). On the Changing Colour of Food & Drink. Int. J. Gastron. Food Sci..

[51] Shanthamma S., Preethi R., Moses J.A., Anandharamakrishnan C. (2021). 4D Printing of Sago Starch with Turmeric Blends: A Study on PH-Triggered Spontaneous Color Transformation. ACS Food Sci. Technol..

[52] Ghazal A.F., Zhang M., Liu Z. (2019). Spontaneous Color Change of 3D Printed Healthy Food Product over Time after Printing as a Novel Application for 4D Food Printing. Food Bioprocess Technol..

[53] Ghazal A.F., Zhang M., Bhandari B., Chen H. (2021). Investigation on Spontaneous 4D Changes in Color and Flavor of Healthy 3D Printed Food Materials over Time in Response to External or Internal PH Stimulus. Food Res. Int..

[54] He C., Zhang M., Guo C. (2020). 4D Printing of Mashed Potato/Purple Sweet Potato Puree with Spontaneous Color Change. Innov. Food Sci. Emerg. Technol..

[55] Chen C., Zhang M., Guo C., Chen H. (2021). 4D Printing of Lotus Root Powder Gel: Color Change Induced by Microwave. Innov. Food Sci. Emerg. Technol..

[56] Wang R., Li Z., Shi J., Holmes M., Wang X., Zhang J., Zhai X., Huang X., Zou X. (2021). Color 3D Printing of Pulped Yam Utilizing a Natural PH Sensitive Pigment. Addit. Manuf..

[57] Chen C., Zhang M., Mujumdar A.S., Phuhongsung P. (2021). Investigation of 4D Printing of Lotus Root-Compound Pigment Gel: Effect of PH on Rapid Colour Change. Food Res. Int..

[58] Guo C., Zhang M., Devahastin S. (2021). Color/Aroma Changes of 3D-Printed Buckwheat Dough with Yellow Flesh Peach as Triggered by Microwave Heating of Gelatin-Gum Arabic Complex Coacervates. Food Hydrocoll..

[59] Zhan J., Fu J., Jin D., Yuan Y., Shen S., Li G., Chen Y. (2023). Surimi Freshness Monitoring of 4D Printing Material with Anthocyanin. J. Food Eng..

[60] Szczesniak A.S. (2002). Texture Is a Sensory Property. Food Qual. Prefer..

[61] Megdich A., Habibi M., Laperrière L. (2023). A Review on 4D Printing: Material Structures, Stimuli and Additive Manufacturing Techniques. Mater. Lett..

[62] Oral M.O., Derossi A., Caporizzi R., Severini C. (2021). Analyzing the Most Promising Innovations in Food Printing. Programmable Food Texture and 4D Foods. Future Foods.

[63] Gao B., Yang Q., Zhao X., Jin G., Ma Y., Xu F. (2016). 4D Bioprinting for Biomedical Applications. Trends Biotechnol..

[64] Fahmy A.R., Kant J., Marius Knorz, Jekle M. 4D Printing of Shape-Morphing Foods: Control of Stimulus-Driven Deformations through Protein-Starch Thermal Transitions. Proceedings of the ICEF14 International Congress on Engineering and Food.

[65] He C., Zhang M., Devahastin S. (2020). Investigation on Spontaneous Shape Change of 4D Printed Starch-Based Purees from Purple Sweet Potatoes As Induced by Microwave Dehydration. ACS Appl. Mater. Interfaces.

[66] Chen F., Zhang M., Liu Z., Bhandari B. (2021). 4D Deformation Based on Double-Layer Structure of the Pumpkin/Paper. Food Struct..

[67] He C., Zhang M., Devahastin S. (2021). Microwave-Induced Deformation Behaviors of 4D Printed Starch-Based Food Products as Affected by Edible Salt and Butter Content. Innov. Food Sci. Emerg. Technol..

[68] Shi Y., Zhang M., Phuhongsung P. (2022). Microwave-Induced Spontaneous Deformation of Purple Potato Puree and Oleogel in 4D Printing. J. Food Eng..

[69] Dick A., Gao Y., Bhandari B., Prakash S. (2021). Influence of Drying Method and 3D Design on the 4D Morphing of Beef Products. Appl. Food Res..

[70] Guo J., Zhang M., Li J., Fang Z. (2023). Using Soy Protein Isolate to Improve the Deformation Properties of 4D-Printed Oat Flour Butterfly. Food Bioprocess Technol..

[71] Nishihara Y., Kakehi Y. (2021). Magashi: Fabrication of Shape-Changing Edible Structures by Extrusion-Based Printing and Baking. Proceedings of the Creativity and Cognition.

[72] Pulatsu E., Su J.W., Lin J., Lin M. (2022). Utilization of Ethyl Cellulose in the Osmotically-Driven and Anisotropically-Actuated 4D Printing Concept of Edible Food Composites. Carbohydr. Polym. Technol. Appl..

[73] Liu Z., He C., Guo C., Chen F., Bhandari B., Zhang M. (2021). Dehydration-Triggered Shape Transformation of 4D Printed Edible Gel Structure Affected by Material Property and Heating Mechanism. Food Hydrocoll..

[74] Jefferson D.R., Lacey A.A., Sadd P.A. (2007). Crust Density in Bread Baking: Mathematical Modelling and Numerical Solutions. Appl. Math. Model..

[75] Anantheswaran R.C., Liu L. (1994). Effect of Viscosity and Salt Concentration on Microwave Heating of Model Non-Newtonian Liquid Foods in a Cylindrical Container. J. Microw. Power Electromagn. Energy.

[76] Lerman M.J., Lembong J., Gillen G., Fisher J.P. (2018). 3D Printing in Cell Culture Systems and Medical Applications. Appl. Phys. Rev..

[77] Rutzerveld C. (2018). Food Futures: How Design and Technology Can Reshape Our Food System.

